# Combination treatment with asiaticoside and rapamycin: A new hope for in-stent restenosis

**DOI:** 10.3892/etm.2013.1155

**Published:** 2013-06-13

**Authors:** TIAN GUO, MING FANG, DADONG ZHANG, XINMING LI

**Affiliations:** 1 Medical School, Tongji University, Shanghai 200092;; 2 Department of Cardiology, Central Hospital of Minhang, Shanghai 201199;; 3 Cardiology Department, Shanghai Pudong New Area Zhoupu Hospital, Shanghai 201318, P.R. China

**Keywords:** in-stent restenosis, asiaticoside, rapamycin, Smad7

## Abstract

The aim of this study was to investigate and characterize the efficacy and mechanism of action of asiaticoside in combination with rapamycin in the inhibition of in-stent restenosis (ISR). The effects of asiaticoside combined with rapamycin on cell proliferation *in vitro* were evaluated by MTT assay. The mRNA expression was analyzed by quantitative polymerase chain reaction (qPCR). Enzyme-linked immunosorbent assay (ELISA) was used to confirm protein synthesis. The cell growth inhibition rate in the combination group was significantly higher compared with those in the asiaticoside and rapamycin groups for human aortic fibroblasts (HAFs; 63.50±3.83, 53.06±8.10 and 60.34±4.9%, respectively) and human aortic smooth muscle cells (HASMCs; 33.12±1.35, 26.21±7.59 and 28.27±4.92, respectively; P<0.05). However, for human coronary artery endothelial cells (HCAECs), the cell growth inhibition rates in the combination, asiaticoside and rapamycin groups were 11.09±1.17, 26.22±4.24 and 34.80±2.80%, respectively (P<0.05), as detected by MTT assay. The qPCR assay showed that in the combination group the level of von Willebrand factor (vWF) mRNA was downregulated, while platelet endothelial cell adhesion molecule (PECAM-1) and endothelial nitric oxide synthase (eNOS) mRNAs were upregulated in HCAECs compared with the rapamycin group (P<0.05). Transforming growth factor (TGF)-β1 and TIMP1 mRNAs were downregulated while Smad7 and matrix metalloproteinase 1 (MMP1) mRNAs were upregulated in HAFs compared with the rapamycin and AT groups (P<0.05). The ELISA showed that the type I collagen level was significantly reduced in HASMCs and HAFs (P<0.05). The data suggest that asiaticoside combined with rapamycin may be effective in the reduction of ISR.

## Introduction

In-stent restenosis (ISR) following vascular intervention affects the long-term curative effect markedly (1). Although drug-eluting stents (DESs) have favorable antiproliferative properties, ISR remains a serious problem which should not be neglected. At present, rapamycin-eluting stents are widely used in the clinic to reduce restenosis. As an immunosuppressive agent, rapamycin addresses the issue of neointimal proliferation, a pathology contributing to restenosis. However, the inhibition of endothelial cells (ECs) induces delayed endothelialization, which increases the risk of in-stent thrombosis.

Asiaticoside is a white needle-like crystalline material, which is a saponin component extracted from *Centella asiatica*, a plant of the Umbelliferae family, which has been used for the treatment of hypertrophic scars for numerous years and is an ingredient in Chinese traditional herbal medicines. Previous studies have demonstrated that asiaticoside has a variety of biological effects, including anti-inflammatory (2) and anti-ulcerative properties (3), tumor cell apoptosis-inducing activity (4), anti-hepatofibrotic (5) and anti-anxiety actions (6), and wound-healing activity (7). It has been reported that asiaticoside may suppress scar formation by inhibiting the proliferation of fibroblasts and extracellular matrix (ECM) synthesis (8). However, the precise pathological mechanism of action of asiaticoside at the molecular and gene expression levels remains unknown.

Transforming growth factor β (TGF-β) belongs to a family of cytokines with a variety of functions relating to fibrosis, growth, differentiation and apoptosis (9). TGF-β is upregulated following coronary angioplasty (10). Several studies have demonstrated the important role of TGF-β in intimal thickening and arterial remodeling, which contribute to ISR (11). Shi *et al* observed that TGF-β1 induces myofibroblast migration as well as arterial remodeling by collagen deposition (12).

TGF-β1 promotes synthesis of the ECM by upregulating the α2 (type I) collagen gene, which results in an increase in the synthesis of type I collagen in fibroblasts; the increased ECM contributes to artery remodeling. The Smad signaling pathway is the primary signaling pathway for TGF-β. Among the Smad family, Smad7 is a general antagonist of the TGF-β family. Smad7 regulates TGF-β signaling via a negative feedback loop and mediates the crosstalk between TGF-β and other signaling pathways (13). Matrix metalloproteinase 1 (MMP1) belongs to the family of MMPs which degrade ECM. TIMP1 is an inhibitor of MMP1. A reduction of the TIMP1/MMP1 ratio value may inhibit the synthesis of collagen (14). von Willebrand factor (vWF), platelet endothelial cell adhesion molecule (PECAM-1) and endothelial nitric oxide synthase (eNOS) are considered to be functional markers of vascular ECs.

## Materials and methods

### 

#### Materials

Bare metal stents (BMSs) were purchased from Shanghai MicroPort Medical (Group) Co., Ltd. (Shanghai, China), asiaticoside was purchased from Guangxi Changzhou Natural Pharmaceutical Co., Ltd., (Nanning, China), rapamycin was obtained from Shanghai Gene Biotechnology company (Shanghai, China), Shandon Excelsior ES™ Tissue Processor was purchased from Thermo Fisher Scientific Inc. (Waltham, MA, USA) and the EXAKT 310 CP Basic cutting system was purchased from Exakt Technologies, Inc. (Oklahoma City, OK, USA). Human aortic fibroblasts (HAFs), human aortic smooth muscle cells (HASMCs) and human coronary artery endothelial cells (HCAECs) were purchased from ScienCell Research Laboratories (Carlsbad, CA, USA). Trypsin 0.25% (w/v), 0.53 mM EDTA, endothelial cell medium, fibroblast medium, smooth muscle cell medium, 3-(4,5-dimethylthiazol-2-yl)-2,5-diphenyltetrazolium bromide (MTT), TRIzol and SuperScript^®^ II were purchased from Invitrogen Life Technologies (Carlsbad, CA, USA). SYBR^®^ Premix Ex Taq™ II (Perfect Real Time) was obtained from Takara Bio, Inc. (Shiga, Japan). ABI PRISM^®^ 7900HT Sequence Detection system was purchased from Invitrogen Life Technologies and the enzyme-linked immunosorbent assay (ELISA) kit for collagen type I was purchased from Shanghai BlueGene Biotech Co., Ltd. (Shanghai, China).

### Methods

#### Cell cultures

Primary HCAECs were cultured in EC growth medium (EBM-2) containing 5% fetal bovine serum (FBS) and 1% endothelial cell growth supplement (ECGS, Cat no. 1052) and 5 ml of penicillin/streptomycin solution (P/S, Cat no. 0503), antibiotics and antimycotics in an incubator with 5% carbon dioxide at 37°C. Primary HASMCs were cultured in Dulbecco’s modified Eagle’s medium (DMEM) containing 10% FBS, 100 U/ml penicillin, 100 U/ml streptomycin and 1 mmol/l L-glutamine in an incubator with 5% carbon dioxide at 37°C. Primary HAFs were cultured in fibroblast medium (FM) containing 20% FBS, 1% fibroblast growth supplement (FGS) and 5 ml 1% penicillin/streptomycin solution (P/S) in an incubator with 5% carbon dioxide at 37°C. All assays were performed on cells at 80–100% confluence, between passages 1 and 2, and were repeated at least 3 times.

#### Asiaticoside and rapamycin treatment

The cells were seeded in 96-well plates with 8,000 cells per well and treated with asiaticoside, rapamycin or both drugs (24 wells for each group) and incubated with 5% carbon dioxide at 37°C for 24 h. For cell viability analysis, the blank group was treated with 1% dimethyl sulfoxide (DMSO), the asiaticoside group was treated with various concentrations of asiaticoside (1×10^−12^, 1×10^−13^, 1×10^−14^, 1×10^−15^ mol/l) and the rapamycin group was treated with various concentrations of rapamycin (1×10^−12^, 1×10^−13^, 1×10^−14^, 1×10^−15^ mol/l). The combination group was treated with asiaticoside and rapamycin, in which the rapamycin concentration was 10^−9^ mol/l and asiaticoside was used in various concentrations (1×10^−12^, 1×10^−13^, 1×10^−14^, 1×10^−15^ mol/l). For qPCR and ELISA analysis, the blank group was treated with 1% dimethyl sulfoxide (DMSO), the asiaticoside group was treated with 10^−5^ mol/l asiaticoside and the rapamycin group was treated with 10^−9^ mol/l rapamycin. The combination group was treated with 10^−5^ mol/l asiaticoside and 10^−9^ mol/l rapamycin. Following treatment with various drugs, the cells were incubated for 48 h at 37°C. The supernatants were harvested and centrifuged for 15 min at 10,656 × g, and then removed and stored at −20°C for ELISA. The cells were harvested for qPCR.

#### Cell viability analysis by MTT assay

Cell viability was detected using an MTT assay. MTT (5 mg/ml) was added to each well. The cells were incubated for one hour and then made soluble with cytolysis solution (10% Triton X-100, 0.1 mmol/l HCl in isopropyl alcohol solution). Absorbance was determined at 570 nm by spectrophotometry.

#### RNA isolation and qPCR

Briefly, total RNA was isolated using TRIzol according to the manufacturer’s instructions. Reverse transcription-generated cDNA was obtained using Superscript^®^ II. For HCAECs, the vWF, PECAM-1 and eNOS mRNAs were detected. For HAFs, the TGF-β1, Smad7, type I collagen, TIMP1 and MMP1 mRNAs were detected. The primer sequences are listed in Table I. SYBR^®^ Premix Ex Taq™ II (Perfect Real Time) was used. The PCR reaction was carried out with the ABI PRISM^®^ 7900HT Sequence Detection system. The samples were analyzed in duplicate. β-actin was used as an internal control. PCR products were separated by electrophoresis in a 2% agarose gel. Densitometry values representing gene expression were first normalized to β-actin expression (calculated as gene densitometry value/β-actin densitometry value).

#### ELISA

The culture supernatants were collected and stored at −20°C. For HASMCs and HAFs, the type I collagen level was determined using an ELISA kit for collagen type I.

#### Statistical analysis

The data were processed using SPSS software (version 14.0 for Windows; SPSS, Inc., Chicago, IL, USA). The results are presented as the mean ± standard error of the mean (SEM). The differences among experimental groups were compared by one-way analysis of variance (ANOVA), and two sets of isolated sample data were checked using a Student’s t-test. P<0.05 was considered to indicate a statistically significant result.

## Results

### 

#### Cell growth inhibitory rate by MTT assay

**Figure 1.**
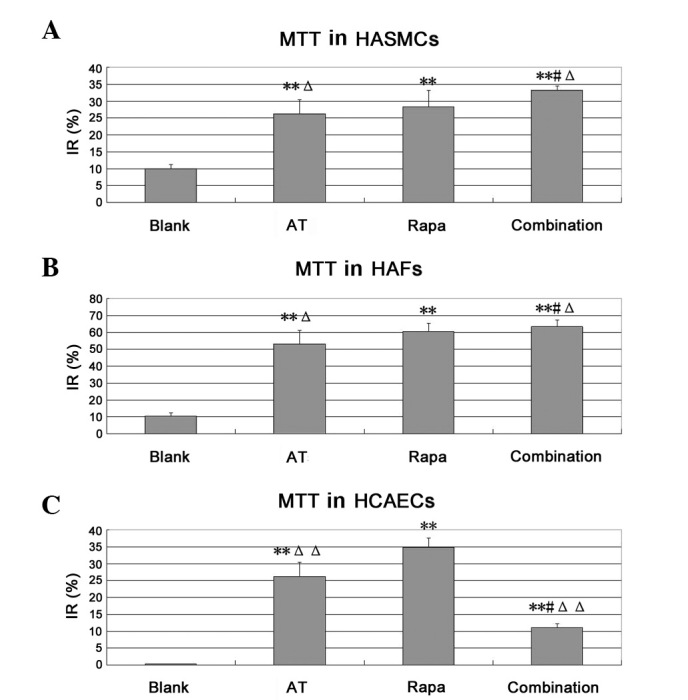
Cell growth inhibitory rate of HCAECs, HASMCs and HAFs, determined by MTT assay. The concentration of asiaticoside (AT) is 10^−5^ mol/l and rapamycin (Rapa) is 10^−9^ mol/l. (A) HASMCs, (B) HAFs and (C) HCAECs. ^**^P<0.01 vs. the blank group, ^#^P<0.05 vs. the asiaticoside group, ^Δ^P<0.05 vs. the rapamycin group, ^ΔΔ^P<0.01 vs. the rapamycin group. IR, inhibitory rate=(1- medication group OD value/control group OD value) ×100. MTT, 3-(4,5-dimethylthiazol-2-yl)-2,5-diphenyltetrazolium bromide; HASMCs, human aortic smooth muscle cells; HAFs, human aortic fibroblasts; HCAECs, human coronary artery endothelial cells.

Compared with the blank group, asiaticoside was able to markedly inhibit the proliferation of HASMCs and HAFs (P<0.01). Compared with the asiaticoside and rapamycin groups, the combination group showed a greater inhibition of HASMCs and HAFs. In HASMCs, the inhibitory rates were 33.12±1.35, 26.21±7.59 and 28.27±4.92%, respectively (P<0.05) and in HAFs, they were 63.50±3.83, 53.06±8.10 and 60.34±4.93%, respectively (P<0.05). These results showed a certain synergism between asiaticoside and rapamycin in HASMCs and HAFs. By contrast, the combination group showed a weaker inhibition of HCAECs compared with that observed in the single drug groups; the inhibitory rates were 11.09±1.17, 26.22±4.24 and 34.80±2.80%, respectively (P<0.05). We suggest that asiaticoside may antagonize the inhibitory effect of rapamycin on vascular ECs (Fig. 1).

**Figure 2.**
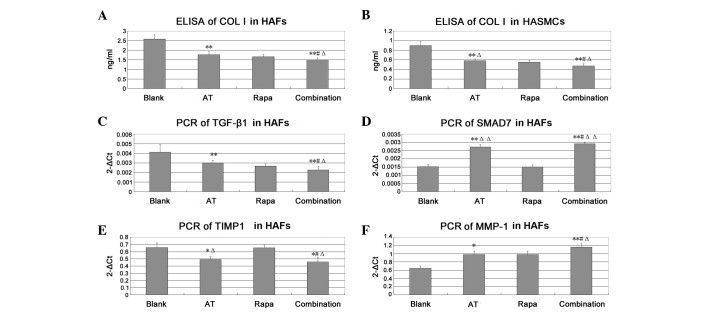
Level of type I collagen in HASMCs and HAFs as shown by ELISA and the levels of TGF-β1, Smad7, MMP1 and TIMP1 in HAFs as shown by qPCR assay. The concentration of asiaticoside (AT) is 10^−5^ mol/l and that of rapamycin (Rapa) is 10^−9^ mol/l. (A and B) ELISA results. (C-F) qPCR results. ^*^P<0.05 vs. the blank group, ^**^P<0.01 vs. the blank group. ^#^P<0.05 vs. the asiaticoside group, ^Δ^P<0.05 vs. the rapamycin group, ^ΔΔ^P<0.01 vs. the rapamycin group. HASMCs, human aortic smooth muscle cells; HAFs, human aortic fibroblasts; ELISA, enzyme-linked immunosorbent assay; qPCR, quantitative polymerase chain reaction; TGF, transforming growth factor; MMP, matrix metalloproteinase.

The levels of type I collagen, TGF-β1, Smad7, MMP1 and TIMP1 are shown in Fig. 2. Asiaticoside significantly reduced the level of type I collagen compared with that in the blank group (P<0.01). The combination treatment was more effective than treatment with asiaticoside or rapamycin alone (P<0.05). Compared with the blank group levels, asiaticoside significantly upregulated Smad7 and MMP1 (P<0.01), but downregulated TGF-β1 and TIMP1 (P<0.01 and P<0.05, respectively). The combination group also showed more effective results than those observed in the asiaticoside and rapamycin groups (P<0.05), suggesting that asiaticoside had a synergism with rapamycin.

#### Levels of vWF, eNOS and PECAM-1 mRNAs in HCAECs as shown by qPCR assay

**Figure 3.**
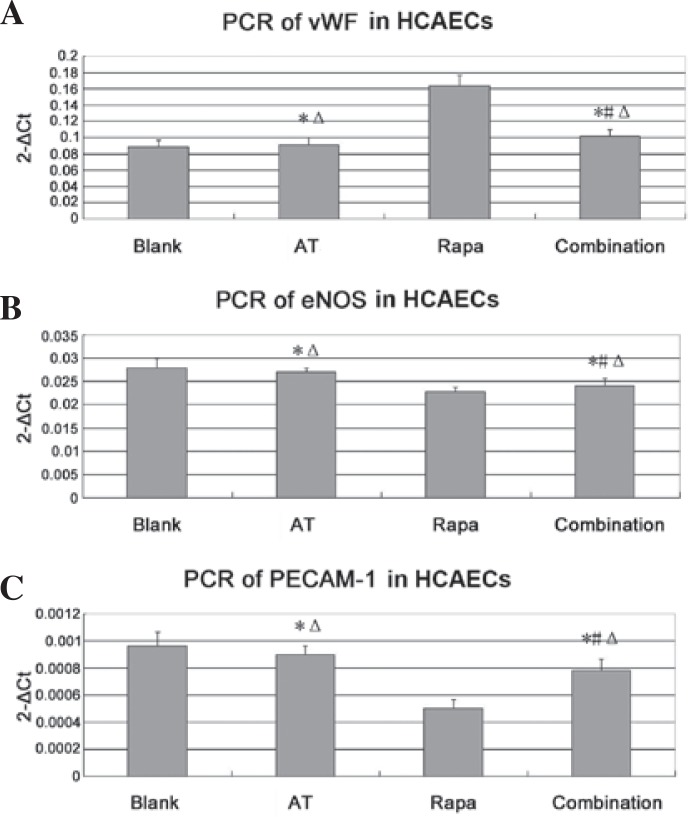
Levels of vWF, eNOS and PECAM-1 mRNAs in HCAECs as shown by qPCR assay. The concentration of asiaticoside is 10^−5^ mol/l and that of rapamycin is 10^−9^ mol/l. mRNAs levels of (A) vWF, (B) eNOS and (C) PECAM-1. ^*^P<0.05 vs. the blank group, ^#^P<0.05 vs. the asiaticoside group, ^Δ^P<0.05 vs. the rapamycin group. vWF, von Willebrand factor; eNOS, endothelial nitric oxide synthase; PECAM-1, platelet endothelial cell adhesion molecule; HCAEs, human coronary artery endothelial cells; qPCR, quantitative polymerase chain reaction.

As shown in Fig. 3, compared with the level in the blank group, the vWF mRNA level of the rapamycin group was significantly increased (P<0.05). The mRNA expression level of the combination group was lower than that of the rapamycin group (P<0.05), indicating that asiaticoside may have antagonized the effect of rapamycin to downregulate the vWF level, and thereby reduced the level of HCAEC apoptosis. The eNOS and PECAM-1 mRNAs levels in the rapamycin group were significantly reduced compared with those in the blank group (P<0.05). However, in the combination group, the levels were higher than those in the rapamycin group (P<0.05), suggesting that asiaticoside may antagonize rapamycin and promote the functional recovery of HCAECs by increasing the levels of eNOS and PECAM-1.

## Discussion

The results indicate that asiaticoside is likely to be effective at reducing ISR *in vivo* and *in vitro*. Asiaticoside combined with rapamycin exerted greater effects than asiaticoside or rapamycin alone. Asiaticoside has a good synergism with rapamycin to inhibit vascular smooth muscle cells (VSMSs) and fibroblasts, while it is also antagonistic to ECs, which may protect the vascular endothelium. The qPCR and ELISA results showed that the combination therapy induced the downregulation of vWF, type I collagen, TGF-β1 and TIMP1, and the upregulation of PECAM-1, eNOS, Smad7 and MMP1. This suggests that the combination therapy may function via the TGF-β pathway.

ISR is a process involving several pathological pathways, in which VSMC and fibroblast proliferation, neointimal formation, negative remodeling of the artery and epithelialization delay play important roles. Rapamycin-eluting stents (RESs) are widely used to treat severe stenosis of the coronary artery. As an immunosuppressant, rapamycin binds to the cytosolic receptor FKBP12, then inhibits mammalian target of rapamycin (mTOR), which leads to inhibition of the down-regulation of the cyclin-dependent kinase inhibitor p27kip1, thereby inhibiting VSMC proliferation and migration (15). However, rapamycin may also inhibit ECs at the same time (16) which contributes to delayed endothelialization. The vascular endothelium is an efficient barrier against thrombosis, lipid uptake and inflammation. In addition, ECs produce various vasoactive substances, which maintain vascular homeostasis (17). Endothelium that has regenerated following percutaneous coronary intervention (PCI) is incompetent in terms of its integrity and function, with poorly formed cell junctions, reduced expression of antithrombotic molecules and reduced nitric oxide production. Delayed endothelial healing, characterized by poor endothelialization, is the primary cause of late and very late stent thrombosis following PCI. One small study demonstrated impaired endothelial function in patients presenting with ISR, compared with matched control subjects. This supports a hypothesis that endothelial dysfunction contributes to the development of restenosis, following PCI (18). Thus, protecting ECs and promoting the recovery of endothelial function requires further study (19).

Our study shows that asiaticoside has a synergism with rapamycin in VSMCs and fibroblasts, which results in a greater increase of cell growth inhibition rate than using a single drug. The combination effects are achieved via several mechanisms. Asiaticoside may inhibit the proliferation of VSMCs and fibroblasts, which is consistent with other studies (8,20,21). Asiaticoside upregulates Smad7 and TGF-β1, thus reducing synthesis of type I collagen. Pan *et al* have demonstrated that asiaticoside inhibits scar fibroblast growth via the Smad signal pathway. The Smad7 protein and mRNA levels were reported to be increased in asiaticoside-treated fibroblasts, compared with control fibroblasts (8,21). It is likely that asiaticoside has different functions in different tissues, and has distinct tissue specificity. Nowwarote *et al* observed that asiaticoside enhanced the expression of type I collagen in human periodontal ligament cells (22), which conflicted with our findings. However, our results in vascular cells are consistent with previous results in scar, wound and renal fibroblasts (23). Our results revealed that the combination reduced the ratio value of TIMP1/MMP1; this may also be reduced by increased TGF-β1 levels, leading to an increase in the degradation of type I collagen.

In ECs, asiaticoside shows significant activity as a rapamycin antagonist, therefore, the inhibition of cell proliferation in the combination group is lower than that in the rapamycin group. There are few studies concerning the effect of asiaticoside on ECs. Zhou *et al* (24) established a rabbit model and observed that asiaticoside had an accelerating action on EC growth and was effective in the prevention of ISR. However, the mechanism remains unclear. vWF is a blood glycoprotein involved in hemostasis. Increased plasma levels are presumed to arise from adverse changes to the endothelium and may contribute to an increased risk of thrombosis. PECAM-1 is a protein which makes up a large portion of endothelial cell intercellular junctions. eNOS is secreted by ECs. Thus, the reduction of vWF mRNA and the increase of PECAM-1 and eNOS mRNAs show that asiaticoside is able to accelerate the recovery of EC function. According to our data, the mechanism may be associated with the enhancement of eNOS and PECAM-1.

## Figures and Tables

**Table I.** Primer sequences used in this study.

Primer	Sequence (5′ to 3′)
vWF forward	GTGGGAAGCTGTAAGTCTGAAGTAG
vWF reverse	CACATCGTTGATGTCAATGGAGTA
PECAM-1 forward	TGAACTCCAACAACGAGAAAATG
PECAM-1 reverse	CCGTAATGACTGTTAGCTTCCATAT
eNOS forward	CGGCATCACCAGGAAGAAGA
eNOS reverse	TCGGAGCCATACAGGATTGTC
TGF-β1 forward	TGGACACGCAGTACAGCAAG
TGF-β1 reverse	GCCCACGTAGTACACGATGG
Smad7 forward	TCATGCAAACTCTTTGGTCGT
Smad7 reverse	TTCTGCTTCCCCTCTTCCTAT
COL1A1 forward	GAGGGCAACAGCCGCTTCAC
COL1A1 reverse	GGAGGTCTTGGTGGTTTTGTATT
TIMP1 forward	GGGCTTCACCAAGACCTACAC
TIMP1 reverse	GGATGGATAAACAGGGAAACACT
MMP1 forward	TGCTCTTTCTGAGGAAAACACT
MMP1 reverse	GCTATCATTTTGGGATAACCTG
β-actin forward	CTGGAACGGTGAAGGTGACA
β-actin reverse	CGGCCACATTGTGAACTTTG

vWF, von Willebrand factor; PECAM-1, platelet endothelial cell adhesion molecule; eNOS, endothelial nitric oxide synthase; TGF, transforming growth factor; COL1A1, type I collagen; MMP, matrix metalloproteinase.
